# Allosteric activation of PI3Kα by oncogenic mutations

**DOI:** 10.18632/oncotarget.913

**Published:** 2013-02-28

**Authors:** John E. Burke, Olga Perisic, Roger L. Williams

**Affiliations:** Medical Research Council, Laboratory of Molecular Biology, Cambridge CB2 0QH, UK; Medical Research Council, Laboratory of Molecular Biology, Cambridge CB2 0QH, UK; Medical Research Council, Laboratory of Molecular Biology, Cambridge CB2 0QH, UK

**Figure 1:**
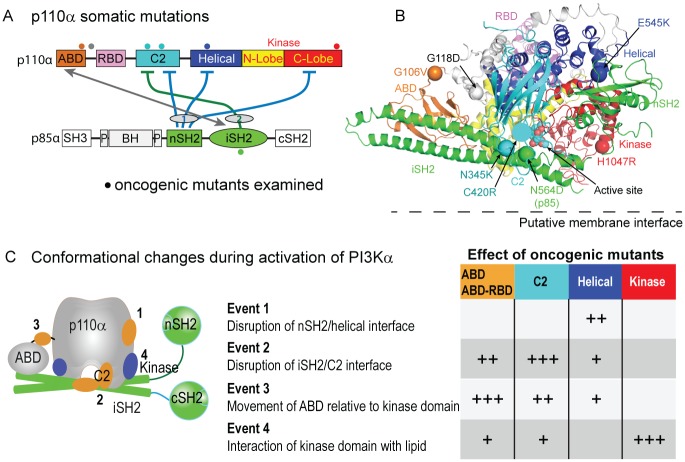
Oncogenic mutants in PI3K A. Domain architecture of PI3Kα and locations of inhibitory contacts between the p110 and p85 subunits. Oncogenic mutants examined by HDX-MS [4] are shown as spheres. B. Crystal structure of the nSH2 and iSH2 of p85 with the p110α catalytic subunit [6]. C. Conformational changes discovered in the catalytic cycle of the WT enzyme, and the effects of oncogenic mutations on these conformational events.

The gene encoding the p110α catalytic subunit of class IA phosphoinositide 3-kinase (*PIK3CA*) is one of the most frequently mutated genes in human cancer [1]. Somatic mutations are widely dispersed throughout every domain of the catalytic subunit (Fig 1A). Two “hot spot” mutants have been shown biochemically and structurally to activate lipid kinase activity by distinct mechanisms [2]. Rare mutations, which have been shown to play a role in endometrial cancers [3], also increase lipid kinase activity. The mechanism of activation of rare mutations that are distant from both the active site and membrane interface have remained ambiguous. We have recently determined a set of allosteric conformational events that occur upon activation of wild type PI3Kα using hydrogen/deuterium exchange mass spectrometry (HDX-MS), and we find that all oncogenic mutants studied activated lipid kinase activity by mimicking or enhancing these specific events [4].

The lipid kinase activity of the class IA PI3Ks is tightly regulated by the p85 regulatory subunit which stabilizes, inhibits, and allows activation downstream of receptor tyrosine kinases (RTKs) (Fig. 1A, 1B). The interaction between the catalytic and regulatory subunit is mediated by the interaction of the adaptor-binding domain (ABD) of p110 and the coiled coil iSH2 domain of p85 [5]. The enzyme is inhibited by contacts between the N-terminal SH2 domain (nSH2) of p85 and the C2, helical and lipid kinase domains of the p110 subunit [5, 6]. The binding of phosphorylated tyrosine residues in RTKs and their associated proteins by the nSH2 disrupts this inhibitory interface and activates the enzyme. The enzyme is also inhibited by contacts between the C2 of p110 and the iSH2 of p85 [7]. The C-terminal SH2 (cSH2) domain of p85 which inhibits the p110δ and p110β isoforms of PI3K has no inhibitory influence on the p110α isoform [8].

To determine the mechanism of activation by oncogenic mutants we first investigated conformational changes that occur in the wild type upon binding to tyrosine-phosphorylated peptides derived from RTKs (pY), and upon binding to phospholipid membranes [4]. We identified four distinct conformational events that occur in the wild type PI3Kα upon activation (Fig. 1C): (i) Disruption of the nSH2/helical interface, (ii) Disruption of the C2/iSH2 interface, (iii) Movement of the ABD relative to the kinase domain, and (iv) Interaction of the lipid kinase domain with membranes.

We propose that these events are involved in the transition of a cytosolic, inhibited form of p110α to an open, activated form on the membrane surface. Oncogenic mutants in the ABD (G106V), ABD-RBD (Ras-binding domain) linker (G118D), C2 (N345K, C420R), and the hot spot mutants in the helical (E545K) and kinase domains (H1047R) upregulated lipid kinase activity by mimicking or enhancing one or more of the conformational events identified in the wild type.

Mutations in the ABD and ABD-RBD linker caused both enhanced movement of the ABD domain relative to the kinase domain, and disruption of the C2/iSH2 interface compared to the WT enzyme. This same relationship was seen in mutations in the C2 domain, which also caused disruption of the C2/iSH2 interface along with movement of the ABD relative to the kinase domain. This shows that these two regions are allosterically linked to each other. These mutations can be activated by RTK derived pY, and are hyperactivated compared to the WT enzyme.

The presence of the helical domain hotspot mutation located at the nSH2/helical interface fully disrupted this contact and made the mutant insensitive to pY activation, as predicted from previous biochemical and structural data [5, 6]. The kinase domain hotspot mutation caused conformational changes in the C-lobe of the kinase domain located at the membrane interface. Interestingly, the regions of the kinase domain that were exposed by this mutation are the exact same regions that were protected by the cSH2 domain in the p110β and p110δ isoforms [8]. This raises an interesting possibility that p110α has acquired inhibitory mutations in the kinase domain that enable it to bypass cSH2 mediated inhibition that occurs in the p110β and p110δ isoforms.

The important role of oncogenic mutants of PI3Kα in the development of a variety of human cancers makes the development of novel inhibitory strategies an important goal. The discovery of these unexpected allosteric conformational changes that are enhanced in oncogenic mutants opens up a novel strategy towards designing small molecule inhibitors that function outside of the conserved ATP binding pocket.
